# Suprapatellar versus infrapatellar approaches in the treatment of tibia intramedullary nailing: a retrospective cohort study

**DOI:** 10.1186/s12891-019-2961-x

**Published:** 2019-11-28

**Authors:** Yiliang Cui, Xingyi Hua, Florian Schmidutz, Jian Zhou, Zongsheng Yin, Shuang G. Yan

**Affiliations:** 1 Department of Orthopaedic Surgery, The First Affiliated Hospital of Anhui Medical University, Hefei, China; 2 Department of Orthopaedic Surgery, Physical Medicine and Rehabilitation, University of Munich (LMU), Munich, Germany; 3 BG Trauma Center, Eberhard Karls University Tübingen, Tübingen, Germany

**Keywords:** Tibial shaft fractures, Intramedullary nail, Infrapatellar, Suprapatellar

## Abstract

**Background:**

Tibial shaft fractures are routinely managed with intramedullary nailing (IMN). An increasingly accepted technique is the suprapatellar (SP) approach. The purpose of this study was to compare the clinical and functional outcomes of knee joint after tibia IMN through an suprapatellar (SP) or traditional infrapatellar (IP) approach.

**Methods:**

Retrospective analysis was performed in patients with tibial shaft fractures that were treated with IMN through a SP or IP approach between 01/01/2014 and 31/12/2016. The clinical and functional outcomes of the knee were assessed with the Hospital for Special Surgery (HSS) Knee Score. Secondary outcomes included the operation time and intraoperative blood loss.

**Results:**

A total of 50 patients/fractures (26 IP and 24 SP) with a minimum follow-up of 15 months were evaluated. All fractures were OTA 42. No significant differences were found between the two groups in age, gender, side of fractures, operation time, intra-operative blood loss, and follow-up time. No significant difference was seen in HSS score (*P* = 0.62) between them. Sub analysis of all the HSS components scores revealed no significant differences between pain (*P* = 0.57), the stand and walk (*P* = 0.54), the need for walking stick (*P* = 0.60) and extension lag (*P* = 0.60). The other HSS components showed full scores (IP 10 vs. SP 10) in both approaches, including muscle force, flexion deformity and stability components. The range of motion (ROM) component score was superior in the IP group (*P* = 0.04) suggesting a higher ROM.

**Conclusions:**

Both SP and IP approach results in equivalent overall HSS knee scores. However, for the HSS component, the IP approach was superior to SP approach regarding the ROM.

## Background

Tibial shaft fractures are primarily caused by high-energy trauma and are the most common long bone fracture seen, with 2% of all fractures occurring in the adults adult [1, 2]. The insertion of an intramedullary nail (IMN) with interlocking screws is reported to be a successful surgical approach for treating tibial shaft fractures and allows for early functional rehabilitation [3, 4]. Traditional infrapatellar approach for tibia IMN is a popular surgical procedure used in the treatment of tibial shaft fractures. However, IMN insertion through infrapatellar (IP) approach remained technically challenging due to quadriceps muscle force resulting in proximal fracture fragments displacement with the knee in flexion, and an increased risk of valgus and procurvatum deformities following tibial nailing [5, 6]. Besides, chronic anterior postoperative knee pain is one of the most frequent complications after IMN insertion, the incidence was reported varying from 10 to 80% [7].

To overcome these issues, the semiextended approach for tibial IMN insertion was first described by Tornetta et al. [8], and later modified to a suprapatellar (SP) approach using a midline quadriceps tendon insertion site by Cole et al. [9]. This new approach suggests that valgus and procurvatum malalignment has been more easily avoided when the knee is maintained in extension and allows for easier anteroposterior and lateral imaging of the tibia [10, 11]. However, the main concern of this approach is the potential for damage to the patellofemoral articulation with a concurrent effect on anterior knee pain after intramedullary nail fixation and patellofemoral arthritis [12]. There is no reliable evidence on the incidence of patellofemoral joint damage, limiting its clinical application. A recent randomized controlled trial (RCT) showed that suprapatellar approach was superior to infrapatellar approach for the treatment of tibial shaft fracture regarding the functional knee outcomes [13]. Nevertheless, several studies showed no significant differences in pain, knee range of motion or knee functional score between the SP and IP approaches [6, 14, 15]. To date, there is no consistent conclusion about whether suprapatellar approach is superior to infrapatrellar approach.

The current study therefore compared the clinical and functional outcomes between SP and IP approaches for nailing a tibial shaft fracture. This retrospective analysis was to determine whether the SP approach was superior to the IP approach with respect to functional knee outcomes.

## Patients and methods

This retrospective study was completed at Trauma Center of the First Affiliated Hospital of Anhui Medical University. Every skeletally mature patient with a tibia fracture who underwent treatment with an intramedullary nail between January 2014 and December 2016 was identified. The study was approved by the Ethics Committee of the First Affiliated Hospital of Anhui Medical University and conducted in accordance with the Helsinki Declaration of 1975 as revised in 2013.

Tibia fractures were graded according to the Orthopaedic Trauma Association Classification (OTA/AO) scheme based on the initial injury films and computed tomography (CT) [16]. Inclusion criteria included extraarticular tibia fractures (OTA). Exclusion criteria included prior fracture of ipsilateral tibia, open fracture, intra-articular tibia fracture, pathological fracture, multiple trauma, and insufficient radiographic or chart data.

Patients were divided into two groups: those treated using a SP IMN insertion technique versus those using IP IMN insertion technique, which included insertion through a medial parapatellar technique. Patient demographics and characteristics are shown in Table 1. Procedures were performed by the same senior orthopaedic surgeon who was well trained in both techniques. All fractures were treated with a reamed IMN in a non-dynamized mode (T2 Tibial Nail, Stryker). All patients were contacted after a minimum of 15 months following surgery, and the knee outcomes of all patients were evaluated by a trained and experienced orthopaedic surgeon using the modified knee-rating system of The Hospital for Special Surgery (HSS) [17]. Perioperative blood loss and time to surgery were extracted from the surgical notes.

**Table 1:** Patient demographics and characteristics [mean (SD)]

	Infrapatellar	Suprapatellar	*P* value
No.patient	26	24	NA
Gender [women / men]	23/ 3	8/ 16	0.063
Age [years]	44.81 (14.19)	41.71 (14.25)	0.336
Right/Left	16/10	13/11	0.598
Operation time [min]	65.69(8.30)	66.38 (8.22)	0.794
Blood loss [ml]	43.21 (7.02)	42.75 (7.38)	0.898
Follow-up in months	23.08 (6.97)	23.92 (7.04)	0.675

*NA* Not applicable

For the conventional IP approach group, a 3 cm incision was performed at the medial side of the patellar tendon and the patellar tendon was retracted to the lateral side to get to the anterior tibia at the junction of the anterior cortex and articular surface. Then the knee was flexed to about 130^°^ to obtain the desired nailing entry point, which was defined as medial to the lateral tibial spine on the anteroposterior view and anterior to the articular margin on the lateral view with the guidance of C-arm. The next steps are standard surgical techniques of IMN insertion.

**Fig. 1:**
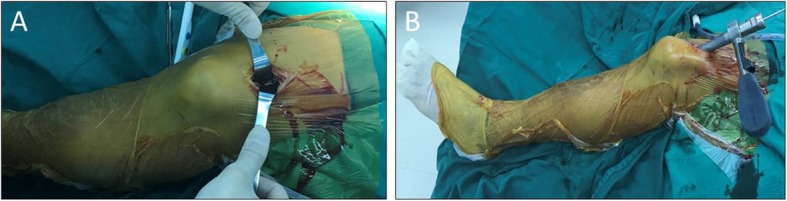
An approximately 3 cm incision is made just above the superior pole of the patella in suprapatellar intramedullary nail (**a**) and the views of specialized cannula system placed in the suprapatellar portal with the knee in the semiextended position (**b**)

**Fig. 2:**
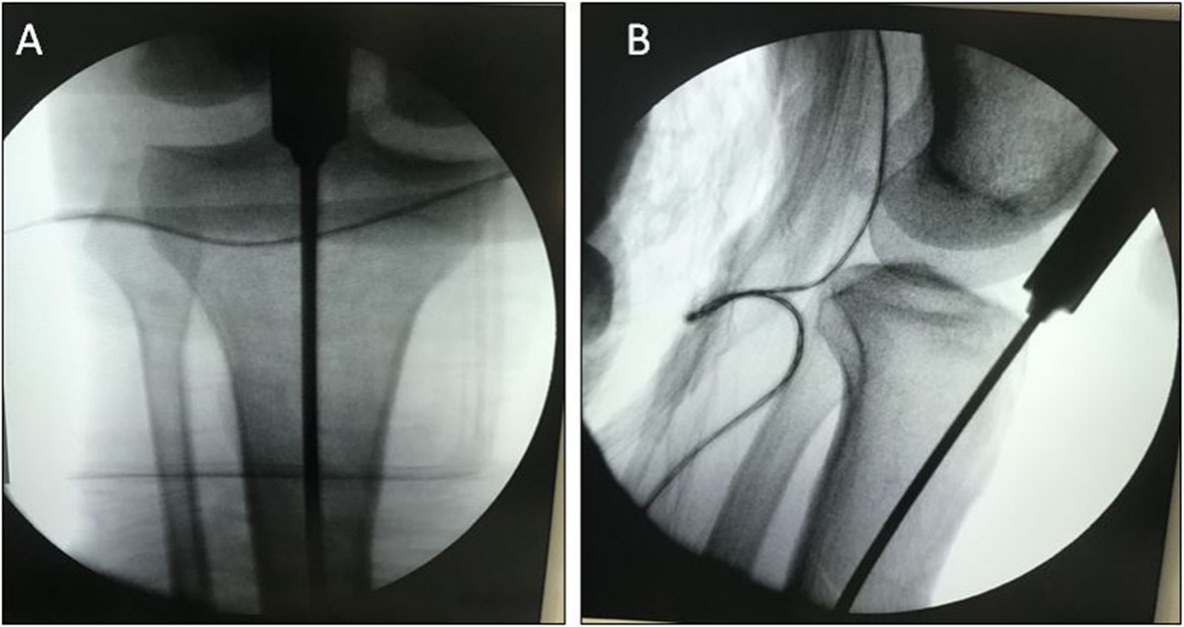
The starting point is established just medial to the lateral tibial spine on the anterior to posterior view (**a**) and at the anterior edge of the tibial plateau on the lateral view (**b**)

For the SP approach group, a 3 cm incision was made approximately 2 cm proximal to the superior pole of the patella (Fig. 1), then the quadriceps tendon and articular capsule were split lengthwise, after which a specialized insertion cannula (T2 Tibial Nail, Stryker) within a protective sleeve was placed at the desired entry point through the trochlear groove under the patellar (Fig. 1). The entry point was defined as the IP approach with the guidance of C-arm (Fig. 2). After that, IMN was inserted through the specialized insertion cannula as per convention.

Data were obtained from the hospital records and the standardized data sheets completed by the clinical teams involved in surgical care. The data collectors received training and supervision from the primary investigators in the identification and classification of complications and process measures. Statistical analyses were performed using SPSS version 19.0 (SPSS, Chicago, IL). Data are given as mean ± standard deviation (SD). After testing for normality with the Kolmogorov-Smirnov test, an unpaired Student’s t-test was used to compare the SP and IP groups regarding the total HSS score and all individual knee function scores of HSS. Chi-square test was applied to compare the difference in complication incidence between different groups. A *p*-value < 0.05 was considered significant.

## Results

A total of 50 patients/fractures were included in this study, 24 SP and 26 IP. All fractures were OTA 42. Patients underwent follow-up for a minimum of 15 months. The socio-demographic data are given in Table 1. No significant differences were found between the two groups in age, gender, side of fractures, operation time, intra-operative blood loss, and follow-up time.

The overall HSS scores were 97.27 and 97.21 for the IP and SP groups, respectively, and they were not statistically different (*P* = 0.62). Sub analysis of all the HSS components scores demonstrated that there were no significant differences between the pain component (IP 28.46 ± 2.75 vs. SP 28.98 ± 2.07; *P* = 0.57) as well as stand and walk component (IP 21.54 ± 2.35 vs. SP 21.54 ± 1.61; *P* = 0.54). The range of motion (ROM) component score was superior in the IP group (IP 17.5 ± 1.03 vs. SP 16.71 ± 1.65; *P* = 0.04) suggesting a higher range of motion. The other HSS components showed full scores (IP 10 vs. SP 10) in both approaches, including muscle force, flexion deformity and stability components. No statistical differences were found in the current study between the two approaches regarding complications, incidence of the need for walking stick (IP 2/26 vs. SP 1/24; *P* = 0.60) and extension lag (IP 2/26 vs. SP 1/24; *P* = 0.60) (Table 2).

**Table 2:** Knee functional outcomes data for infrapatellar and suprapatellar intramedullary tibial nails [mean (SD)]

	Infrapatellar	Suprapatellar	*P* value
HSS score	97.27 (5.67)	97.21 (4.98)	0.622
Pain score	28.46 (2.75)	28.98 (2.07)	0.574
Stand&Walk score	21.54 (2.35)	21.54 (1.61)	0.540
Range of motion score	17.5 (1.03)	16.71 (1.65)	0.041
Muscle force score	10	10	NA
Flexion deformity score	10	10	NA
Stability score	10	10	NA
Need for walking stick (or not)	2 (24)	1 (23)	0.600
Extention lag (or not)	2 (24)	1 (23)	0.600
Varus&valgus (or not)	0 (26)	0 (24)	NA

NA: Not applicable.

## Discussion

This study provides the clinical rationale that the SP and IP approaches can get similar knee functional outcomes in the treatment of tibial shaft fracture. The results demonstrated that the SP approach is comparable to the traditional IP technique with regard to the overall HSS knee score. However, for the HSS component, our study showed that the IP approach was superior to SP approach with respect to the range of motion.

Evaluation of the overall HSS knee score of SP and IP approaches for the treatment of tibial shaft fracture in this study demonstrated comparable outcomes. Our results are in line with the previous data reporting comparable functional knee outcomes between SP and IP approaches [14, 15]. Chan DS et al. compare the clinical outcomes of the knee joint after SP versus IP tibial nail insertion in a prospective randomized study with 42 patients and 12 months of follow-up, and reported no significant difference regarding the Lysholm knee scores [14]. Courtney PM et al. similarly evaluated 24 patients who underwent IP IMN and 21 patients who underwent SP IMN after more than 8 months, and found similar results regarding the Oxford Knee Score [15]. However, a meta-analysis of RCTs indicates that SP IMN has higher Lysholm knee scores than IP IMN [18]. Similarly, Sun Q et al. found a higher Lysholm knee score in SP IMN than IP IMN in a prospective randomized study with 162 patients after a mean of 2 years [13]. However, it must be noticed that type of knee functional score in the previous study was different from the present study, which may generate heterogeneity.

In accordance with other studies, the present study revealed comparable operation time and blood loss between SP IMN and IP IMN. These findings are confirmed by Sun Q et al. who reported a mean operation time of 71.01 min and an intraoperative blood loss of 22.11 ml for SP IMN, showing no difference compared to IP IMN with operation time of 73.26 min and intraoperative blood loss of 21.67 ml. Chen X et al. conducted a meta-analysis of RCTs and reported that there were no significant differences in the operative time and blood loss between SP and IP groups [19]. However, review analysis of RCTs indicated that SP IMN could significantly reduce total blood loss compared to IP IMN [20]. The difference might be explained by differing surgical techniques, especially during preparation or insertion of the nails and screws.

The HSS pain component demonstrated that there was no significant difference between SP and IP groups. This finding goes along with a prospective randomized study operated by Chan DS et al. [14] who compared the visual analog score (VAS) of SP to IP approaches with 42 patients, and reported no significant difference regarding VAS pain scores. Similarly, Both Jones et al. [6] and Courtney et al. [15] found that the VAS pain score of the SP group was equivalent to the IP group. However, multiple studies revealed less postoperative knee pain in SP than IP approaches [13, 20, 21]. Sun Q et al. conducted a 2-year follow-up of 162 patients in an RCT study and found lower VAS pain scores following SP IMN compared to IP IMN [13]. The etiology of anterior knee pain is undoubtedly multifactorial, which may be related to cartilage injury, patellar ligament injury, iatrogenic damage to the IP nerve, and the protruding nail end at the tibial plateau [22–24]. Zamora et al. [25] and Gaines et al. [23] conducted cadaveric studies and found that the SP approach for tibial nailing has a similar rate of soft tissue damage compared to the IP approach. These results might interpret the equal pain score in SP and IP groups in the present study.

This study demonstrated that the ROM component was significantly superior in the IP approach. However, the previous studies showed different results compared to our studies [13, 14]. Sun Q et al. reported on a 2-year follow-up after SP tibial IMN insertion in an RCT study with 162 patients and found equivalent knee ROM compared to IP IMN [13]. Chan DS et al. reported no significant differences in knee ROM between SP and IP IMN after 12 months of follow up in an RCT study with 42 patients [14]. Song et al. found that the knee ROM was significantly associated with the severity of knee pain [22]. Moreover, Aksahin et al. [26] found that the damage to quadriceps might worsen the patellar tilt due to the sagittal patellar tilt and quadriceps hypotrophy after tibial nailing, besides, the displacement of patella might have a negative impact on the knee function. Therefore, much attention must be paid to minimize the damage to quadriceps, especially in SP IMN insertion.

Further limitations of this study should be acknowledged. First, this was a retrospective evaluation for comparing the SP and IP approaches in intramedullary nailing of tibia with a small sample size. A long-term RCT study with a larger scale is needed to further evaluate the efficiency of the SP approach. Second, No arthroscopy examination was conducted to identify the cartilage changes pre-operatively and at final follow-up. Third, the alignment of fracture reduction, the fluoroscopy time and the accuracy of the entry point were not evaluated. Further workup in terms of biomechanical stability as well as finite element analysis is necessary to compare the different fixation methods and also compare the results to the practical applicability in the clinical routine.

## Conclusions

Based on the clinical outcomes of SP and IP tibial IMN insertion obtained in this study, the SP and IP approaches can get similar knee functional outcomes in the treatment of tibial shaft fracture with regard to the overall HSS knee score. However, for the HSS component, the IP approach was superior to SP approach regarding the range of motion. A larger prospective trial with long-term follow-up is needed to improve statistical power and establish if any late sequelae exist.

## Data Availability

The datasets analyzed during the current study are available from the corresponding author upon reasonable request.

## Abbreviations

HSS: Hospital for Special Surgery
IMN: Intramedullary nailing
IP: Infrapatellar
OTA: Orthopaedic Trauma Association
RCT: Randomized controlled trial
ROM: Range of motion
SD: Standard deviation
SP: Suprapatellar

## References

[1] Court-Brown CM, Caesar B (2006). Epidemiology of adult fractures: a review. Injury.

[2] Larsen P, Lund H, Laessoe U, Graven-Nielsen T, Rasmussen S (2014). Restrictions in quality of life after intramedullary nailing of tibial shaft fracture: a retrospective follow-up study of 223 cases. J Orthop Trauma.

[3] Vallier HA, Cureton BA, Patterson BM (2011). Randomized, prospective comparison of plate versus intramedullary nail fixation for distal tibia shaft fractures. J Orthop Trauma.

[4] Bone LB, Johnson KD (1986). Treatment of tibial fractures by reaming and intramedullary nailing. J Bone Joint Surg Am.

[5] Hiesterman TG, Shafiq BX, Cole PA (2011). Intramedullary nailing of extra-articular proximal tibia fractures. J Am Acad Orthop Surg.

[6] Jones M, Parry M, Whitehouse M, Mitchell S (2014). Radiologic outcome and patient-reported function after intramedullary nailing: a comparison of the retropatellar and infrapatellar approach. J Orthop Trauma.

[7] Lefaivre KA, Guy P, Chan H, Blachut PA (2008). Long-term follow-up of tibial shaft fractures treated with intramedullary nailing. J Orthop Trauma.

[8] Tornetta P, Collins E (1996). Semiextended position of intramedullary nailing of the proximal tibia. Clin Orthop Relat Res.

[9] Cole JD (2006). Distal tibia fracture: opinion: intramedullary nailing. J Orthop Trauma.

[10] Franke J, Hohendorff B, Alt V, Thormann U, Schnettler R (2016). Suprapatellar nailing of tibial fractures-indications and technique. Injury.

[11] Zelle BA, Boni G (2015). Safe surgical technique: intramedullary nail fixation of tibial shaft fractures. Patient Saf Surg.

[12] Hernigou P, Cohen D (2000). Proximal entry for intramedullary nailing of the tibia. The risk of unrecognised articular damage. J Bone Joint Surg Brit Vol.

[13] Sun Q, Nie X, Gong J, Wu J, Li R, Ge W, Cai M (2016). The outcome comparison of the suprapatellar approach and infrapatellar approach for tibia intramedullary nailing. Int Orthop.

[14] Chan DS, Serrano-Riera R, Griffing R, Steverson B, Infante A, Watson D, Sagi HC, Sanders RW (2016). Suprapatellar versus Infrapatellar Tibial nail insertion: a prospective randomized control pilot study. J Orthop Trauma.

[15] Courtney PM, Boniello A, Donegan D, Ahn J, Mehta S (2015). Functional Knee Outcomes in Infrapatellar and Suprapatellar Tibial Nailing: Does Approach Matter?. Am J Orthop (Belle Mead, NJ).

[16] Marsh JL, Slongo TF, Agel J, Broderick JS, Creevey W, DeCoster TA, Prokuski L, Sirkin MS, Ziran B, Henley B (2007). Fracture and dislocation classification compendium - 2007: Orthopaedic trauma association classification, database and outcomes committee. J Orthop Trauma.

[17] Leung KS, Shen WY, So WS, Mui LT, Grosse A (1991). Interlocking intramedullary nailing for supracondylar and intercondylar fractures of the distal part of the femur. J Bone Joint Surg Am.

[18] Gao Z, Han W, Jia H (2018). Suprapatellar versus infrapatellar intramedullary nailing for tibal shaft fractures: a meta-analysis of randomized controlled trials. Medicine.

[19] Chen X, Xu HT, Zhang HJ, Chen J (2018). Suprapatellar versus infrapatellar intramedullary nailing for treatment of tibial shaft fractures in adults. Medicine.

[20] Yang L, Sun Y, Li G (2018). Comparison of suprapatellar and infrapatellar intramedullary nailing for tibial shaft fractures: a systematic review and meta-analysis. J Orthop Surg Res.

[21] Wang C, Chen E, Ye C, Pan Z (2018). Suprapatellar versus infrapatellar approach for tibia intramedullary nailing: A meta-analysis. Int J Surg (London, England).

[22] Song SY, Chang HG, Byun JC, Kim TY (2012). Anterior knee pain after tibial intramedullary nailing using a medial paratendinous approach. J Orthop Trauma.

[23] Gaines RJ, Rockwood J, Garland J, Ellingson C, Demaio M (2013). Comparison of insertional trauma between suprapatellar and infrapatellar portals for tibial nailing. Orthopedics.

[24] Tahririan MA, Ziaei E, Osanloo R (2014). Significance of the position of the proximal tip of the tibial nail: an important factor related to anterior knee pain. Adv Biomed Res.

[25] Zamora R, Wright C, Short A, Seligson D (2016). Comparison between suprapatellar and parapatellar approaches for intramedullary nailing of the tibia. Cadaveric study. Injury.

[26] Aksahin E, Yilmaz S, Karasoy I, Duran S, Yuksel HY, Dogan O, Yildirim AO, Bicimoglu A (2016). Sagittal patellar tilt and concomitant quadriceps hypotrophy after tibial nailing. Knee Surg Sports Traumatol Arthrosc.

